# Cost-effectiveness of cognitive–behavioural therapy for sleep disorder added to usual care in patients with schizophrenia: the BEST study

**DOI:** 10.1192/bjo.2018.2

**Published:** 2018-04-19

**Authors:** Apostolos Tsiachristas, Felicity Waite, Daniel Freeman, Ramon Luengo-Fernandez

**Affiliations:** Health Economics Research Centre, Nuffield Department of Population Health, University of Oxford, UK; Department of Psychiatry, University of Oxford, UK; Health Economics Research Unit, Nuffield Department of Population Health, University of Oxford, UK

## Abstract

**Background:**

Sleep problems are pervasive in people with schizophrenia, but there are no clinical guidelines for their treatment. The Better Sleep Trial (BEST) concluded that suitably adapted cognitive–behavioural therapy (CBT) is likely to be highly effective, although its cost-effectiveness is unknown.

**Aims:**

To assess the potential cost-effectiveness of CBT for sleep disorders in patients with schizophrenia.

**Method:**

An economic evaluation of the BEST study with a 6-month time horizon was used to establish the cost-effectiveness of CBT plus usual care in terms of costs per quality-adjusted life year (QALY) gained. Uncertainty was displayed on cost-effectiveness planes and acceptability curves. Value of information analysis was performed to estimate the benefits of obtaining further evidence.

**Results:**

On average, the treatment led to a 0.035 QALY gain (95% CI −0.016 to 0.084), and £1524 (95% CI −10 529 to 4736) and £1227 (95% CI −10 395 to 5361) lower costs from National Health Service and societal perspectives, respectively. The estimated value of collecting more information about the effects of the CBT on costs and QALYs was approximately £87 million.

**Conclusions:**

CBT for insomnia in people with schizophrenia is effective and potentially cost-effective. A larger trial is needed to provide clear evidence about its cost-effectiveness.

**Relevance:**

Patients with schizophrenia have multiple complex health needs, as well as very high rates of depression, suicidal ideation and poor physical health. The results of this study showed that treating pervasive sleep problems in this patient group with cognitive–behavioural therapy (CBT) is very likely to improve patient quality of life in the short term. Clinicians most commonly use hypnotic medication to treat sleeping disorders. This study indicates that CBT may be an effective and cost-effective intervention in this patient group. This alternative would also be aligned with patient preferences for psychological and behavioural-type therapy.

**Declaration of interest:**

None.

Schizophrenia is among the top ten disorders in terms of burden of illness, disability, and societal and health costs worldwide.^1^ In England, there are approximately 8256 new cases of schizophrenia each year^2^ and the total annual cost of schizophrenia to society is £11.8 billion.^3^ In addition, life expectancy is 15 years shorter for people with these problems.^3^^,^^4^ Sleep problems are pervasive and complex in patients with schizophrenia and adversely affect physical and mental health.^5^ These problems typically comprise a mixture of insomnia, hypersomnia, circadian rhythm disorders and nightmares, and their evidence-based treatment in schizophrenia has been overlooked.^6^ However, how to treat such sleep disturbance in the context of schizophrenia is not the topic of any guideline. The effectiveness of cognitive–behavioural therapy (CBT) in treating sleep problems in patients with schizophrenia was only recently assessed in the Better Sleep Trial (BEST).^7^ The findings from this pilot randomised controlled trial suggest that CBT for insomnia, suitably modified for this population, is likely to be highly effective for improving sleep in patients with schizophrenia. However, given that healthcare resources in mental health are particularly scarce,^8^ it is important that any new intervention, particularly one delivered face-to-face to patients, provides good value for money. As a result, the aim of this study was to use data from BEST to assess the potential cost-effectiveness of CBT in treating sleep disorders in patients with schizophrenia.

## Method

### Study design and patients

We carried out an economic evaluation alongside BEST to establish the cost-effectiveness of CBT in addition to usual care to treat sleeping disorders in patients with schizophrenia. BEST was a prospective, assessor-blind, randomised controlled pilot study in Oxfordshire, Buckinghamshire and Northamptonshire, England. The study recruited 50 patients with persistent, distressing delusions or hallucinations in the context of schizophrenia adult mental health services. Details of the trial and the clinical findings have been reported elsewhere.^7^ The economic evaluation was conducted from National Health Service (NHS) and societal perspectives, complying with guidance for health technology appraisals issued by the National Institute for Health and Care Excellence (NICE) in England.^9^ Outcomes and costs were assessed at baseline, and at 12 weeks and 24 weeks post-treatment. The trial flowchart is presented in Appendix 1.

### Intervention and comparator

The CBT intervention included approximately eight sessions over 12 weeks provided one-to-one by clinical psychologists either in NHS clinics or at patients' homes under weekly clinical supervision by a consultant clinical psychologist. Telephone calls and text messages between sessions were used to maintain treatment momentum. The intervention was written in a manual to guide the work, which was shared with the patient. The principal therapeutic techniques included stimulus control (i.e. learning to associate bed with sleep) and improvement of daytime activity levels. Adaptations needed for the particular problems of delusions and hallucinations interfering with sleep, attempts to sleep being overused by patients as an escape from voices, extensive disruption of circadian rhythms, insufficient daytime activity and fear of the bed based on past adverse experiences were incorporated. Standard care was delivered according to national and local service protocols and guidelines and mainly consisted of antipsychotic medication and contact with the local clinical team.

### Outcomes and costs

Health-related quality of life was measured during the follow-up period using the EuroQol 5 Dimensions 5 Levels (EQ-5D-5L) questionnaire. The five-level version of the EQ-5D-5L questionnaire was preferred over the three-level version, because it is considered to be more sensitive for capturing the health-related quality of life of a relatively young population with mental conditions. EQ-5D-5L responses were then valued using population preferences^10^ and combined with survival information to calculate quality-adjusted life years (QALYs).

Costs were measured with a modified version of the Client Service Receipt Inventory.^11^ The recall period in this questionnaire was 6 months at baseline, and 3 months in each of the two follow-up measurements (hence covering the 6 months after baseline). The health and social care costs included were: admissions to hospital; psychiatrist and other consultant visits; general practitioner and other primary healthcare visits, either at home or at the general practice; visits to and by community psychiatric nurses; visits to any counsellor or therapist; social worker visits; visits to any day care centre; and antipsychotic medicines. The intervention costs were collected for each patient included in the treatment group and included the time taken for the psychologist to deliver the sessions, travelling time to home visits, additional contact time via phone and emails, time for administration and travelling costs. The broader societal perspective also included the costs of informal caregiving (i.e. hours of unpaid care delivered to patients by their family and social environment) and justice services (i.e. contacts with police, nights spent in a police cell or prison, and criminal or civil court appearances). All costs were valued based on unit cost prices derived from different resources and reported in 2014/2015 British pounds (£). The costs of informal caregiving were calculated based on the national minimum hourly wage. A list of the unit costs and their sources is presented in Appendix Tables 2.1 and 2.3.

### Statistical analysis

In a descriptive analysis, we used *t*-tests to obtain mean differences (and their 95% confidence intervals) in age, EQ-5D-5L utilities and costs between patients in the CBT group and patients in the standard care alone group. We performed the same descriptive analysis using Mann–Whitney tests to cross-check statistical certainty. Five patients in the CBT group and two patients in the standard care alone group had missing observations in EQ-5D-5L utilities and/or costs on one or more measurement points. The pattern of missing observations is presented in Appendix Table 2.2. The main analysis included only complete cases (i.e. it excluded seven patients with incomplete information on costs and/or EQ-5D-5L utilities).

To address uncertainty in the results due to the small sample size of the pilot trial, we performed bootstrapping with replacement. For each of the 10 000 bootstrapped samples, we estimated the mean and mean differences in costs and QALYs. The statistical analysis was performed in STATA13.

### Cost-utility and value of information analysis

An incremental analysis was performed, assessing the differences in costs between the two interventions and dividing by the difference in QALYs gained in order to generate an incremental cost-effectiveness ratio (ICER). ICERs were estimated using both an NHS and a societal perspective. The 10 000 bootstrapped pairs of incremental costs and incremental QALYs were plotted on cost-effectiveness planes to display the uncertainty in the estimated ICER. Cost-effectiveness acceptability curves (CEACs) were also derived to display the probability of the CBT treatment being cost-effective, as the ceiling ratio for the maximum acceptable cost-effectiveness ratio varies from £0 to £85 000 per QALY gained.

BEST was designed to inform the design of a larger trial in order to gain more information about the effectiveness and cost-effectiveness of the CBT treatment for sleeping disorders. Therefore, we followed NICE guidance and estimated the expected value of perfect information (EVPI), which represents the monetary value of eliminating the uncertainty in the cost-utility results.^12^ In other words, it provides decision makers with the value of acquiring further information on costs and outcomes for a number of people who may benefit from the additional research. EVPI can potentially be used to set research priorities. If the EVPI exceeds the cost of collecting more information about the effects of an intervention on costs and outcomes, then a further study for this purpose is considered to be worthwhile. In this analysis, we assumed that 10 000 patients would be affected by the treatment per year, based on a report about schizophrenia.^2^ We also assumed that the time frame over which the additional information could be expected to retain its usefulness (i.e. before the intervention becomes obsolete from newer interventions) would be 10 years. This time frame is frequently used in EVPI studies.^13^ Using a discount rate of 3.5%, as suggested in NICE guidance, the effective (discounted) population over a 10-year period was calculated to be 86 077 patients. This population was used in the value of information analysis.

### Sensitivity analyses

Four sensitivity analyses were performed to address the effects of missing data and the difference in costs between the treatment arms at baseline on the cost-utility results. In the first, multiple imputation with predictive mean matching (with three closest observations) was performed to impute missing observations in costs and QALYs during the follow-up period based on costs and EQ-5D-5L utilities at baseline. The imputation process was partitioned by treatment arm and generated 500 imputed data-sets for each missing observation. The data-set with the imputed values was then used in the bootstrapping process, similar to the statistical analysis performed in the main analysis. In the second sensitivity analysis, we used generalised linear models to estimate 6-monthly mean costs (using gamma distribution and log link) and QALYs (using normal distribution and identity link) in the two groups and their differences, adjusted for costs or EQ-5D-5L utilities at baseline. The choice of family distribution and link function in the statistical models were based on the modified Park test and Link test, respectively.

Propensity score matching (PSM) was used in the third sensitivity analysis to match patients in the treatment arms based on their costs and EQ-5D-5L utility at baseline. Local linear regression matching was chosen as it outperformed other PSM techniques (including Mahalanobis, one-to-one, *k*-to-one, kernel, spline and inverse probability weighting) based on percentage standardised bias and variance ratio. In this analysis, all complete cases were included and PSM was performed in each of the 10 000 bootstrapped samples before estimating incremental costs and QALYs. In the last sensitivity analysis, antipsychotic medication was excluded from the costs. This is because other types of mental health medicines were poorly recorded in the questionnaires, and the inclusion of only antipsychotic medicines could have introduced biases in the results.

## Results

### Descriptive analysis

There were 19 patients in the CBT group and 24 patients in the standard care alone group with complete information on costs and EQ-5D-5L utilities. As Table 1 shows, patients in the CBT group were on average 40 years old and the majority were male (63%) and unemployed (84%). Their mean EQ-5D-5L utility was 0.58, which is relatively low compared with the 0.81 national average EQ-5D-5L utility of 40-year-olds in England. There were no statistically significant differences between the two groups at baseline. The mean EQ-5D-5L utility was higher in the CBT group at 12- and 24-week follow-up, but these differences were not statistically significant. More details on patient characteristics at baseline are provided eslewhere.^7^

**Table 1:** Baseline demographics and EQ-5D-5L utilities

	CBT group (*n* = 19)	Standard care alone group (*n* = 24)	Difference
	Mean (s.d.)	Mean (s.d.)	Mean (95% CI)
Age, years	40.4 (12.7)	42.9 (13.4)	−2.5 (−10.7 to 5.6)
Female, *n* (percentage)	7 (37)	8 (33)	^a^
Unemployed, *n* (percentage)	16 (84)	21 (88)	^b^
EQ-5D-5L utility			
Baseline	0.582 (0.208)	0.597 (0.214)	−0.015 (−0.149 to 0.119)
12-week follow-up	0.672 (0.245)	0.552 (0.223)	0.120 (−0.025 to 0.265)
24-week follow-up	0.644 (0.231)	0.566 (0.200)	0.078 (−0.055 to 0.211)

Mann–Whitney tests showed no statistically significant differences in mean scores.

CBT, cognitive–behavioural therapy

a. Pearson χ^2^(1) = 0.0575, Pr = 0.811.

b. Pearson χ^2^(5) = 4.1504, Pr = 0.528.

For the provision of the CBT, a psychologist spent on average 23 h per patient for treatment, administration tasks and travelling to home visits. The intervention costs were on average £490 per patient. For the detailed cost calculation, please see Appendix Table 2.4.

Table 2 presents the 6-month costs before and after randomisation and the differences between the two groups. Compared with the standard care alone group, patients in the CBT group had on average £1462 higher healthcare costs in the 6 months before randomisation, mainly owing to higher day hospital costs (i.e. £851) and higher hospital admission costs (i.e. £414). In the 6 months after randomisation, patients in the CBT group had on average £1532 lower healthcare costs than patients in the standard care alone group. The mean differences in total healthcare costs before and after randomisation were mainly driven by the mean differences in day hospital costs, from £851 higher in the CBT group to £2241 higher in the standard care alone group. It should be noted that the −£2241 difference in mean NHS costs after randomisation incorporates the £490 intervention costs in the treatment group. The difference in total costs from the societal perspective between the two groups decreased from £1055 to −£1236.

**Table 2:** Observed costs over 24 weeks before and after randomisation

	CBT group (*n* = 19)	Standard care alone group (*n* = 24)	Difference
	Mean (s.d.)	Mean (s.d.)	Mean (95% CI)
Hospital admission			
24 weeks before	2402 (2182)	1988 (1928)	414 (−5463 to 6290)
24 weeks after	619 (547)	374 (243)	244 (−880 to 1368)
Hospital visit to psychiatrist			
24 weeks before	111 (25)	326 (130)	−215 (−513 to 84)
24 weeks after	137 (27)	284 (98)	−147 (−376 to 82)
Hospital visit to other doctor			
24 weeks before	222 (180)	19 (11)	204 (−120 to 528)
24 weeks after	199 (120)	88 (38)	111 (−122 to 344)
Day hospital			
24 weeks before	3527 (3093)	2676 (1860)	851 (−6121 to 7823)
24 weeks after	1249 (1211)	3490 (3490)	−2241 (−10 473 to 5991)
General practitioner			
24 weeks before	133 (40)	134 (50)	−1 (−136 to 134)
24 weeks after	150 (40)	146 (30)	4 (−94 to 103)
Another doctor outside hospital			
24 weeks before	130 (120)	53 (32)	76 (−150 to 303)
24 weeks after	10 (7)	23 (11)	−13 (−42 to 15)
Community psychiatric nurse			
24 weeks before	312 (96)	598 (125)	−287 (−619 to 46)
24 weeks after	282 (69)	501 (104)	−219 (−487 to 50)
Counsellor/therapist			
24 weeks before	183 (82)	71 (40)	113 (−61 to 286)
24 weeks after	31 (24)	8 (6)	24 (−22 to 69)
Social worker			
24 weeks before	98 (49)	49 (23)	48 (−55 to 151)
24 weeks after	158 (57)	89 (47)	69 (−79 to 217)
Day care centre			
24 weeks before	144 (79)	76 (50)	68 (−114 to 250)
24 weeks after	151 (92)	82 (49)	69 (−128 to 267)
Antipsychotic medication			
24 weeks before	711 (422)	521 (98)	190 (−598 to 979)
24 weeks after	663 (394)	451 (87)	212 (−521 to 945)
Total NHS costs			
24 weeks before	7972 (3609)	6510 (2709)	1462 (−7470 to 10 395)
24 weeks after	4003^a^ (1540)	5535 (3756)	−1532 (−10 514 to 7450)
Justice costs			
24 weeks before	0 (0)	544 (434)	−544 (−1531 to 442)
24 weeks after	378 (219)	54 (38)	324 (−79 to 726)
Informal care costs			
24 weeks before	154 (143)	17 (5)	136 (−120 to 393)
24 weeks after	27 (11)	48 (11)	−21 (−52;10)
Total societal costs			
24 weeks before	8126 (3611)	7071 (2717)	1055 (−7893 to 10 002)
24 weeks after	4421 (1734)	5657 (3757)	−1236 (−10 331 to 7860)

Non-parametric tests also showed no statistically significant differences between the two groups; the recall period used to calculate costs at baseline was 24 weeks.

CBT, cognitive–behavioural therapy; NHS, National Health Service

a. Including intervention costs.

### Results of the cost-utility analysis

The results of the economic evaluation of the pilot study showed that the treatment leads on average to a 0.035 QALY gain (95% CI −0.016 to 0.084), as well as £1524 (95% CI −10 529 to 4736) and £1227 (95% CI −10 395 to 5361) lower costs from the NHS and societal perspectives, respectively (Table 3). Thus, these results indicate that CBT is likely to have higher benefits at lower costs.

**Table 3:** Estimated costs and QALYs

	CBT group	Standard care alone group	Incremental	ICER
	Mean (95% CI)	Mean (95% CI)	Mean (95% CI)	
Costs: NHS perspective	4003 (1665 to 7414)	5526 (1413 to 13 745)	−1524 (−10 529 to 4736)	Dominant
Costs: societal perspective	4420 (1787 to 8253)	5657 (1511 to 13 863)	−1227 (−10 395 to 5361)	Dominant
QALYs	0.296 (0.254 to 0.336)	0.262 (0.233 to 0.292)	0.035 (−0.016 to 0.084)	

These are the mean estimates from 10 000 bootstrapped samples adjusted for baseline costs or EQ-5D-5L utilities. For this reason, the costs reported here do not correspond with the observed unadjusted costs reported in Table 3.

CBT, cognitive behavioural therapy; NHS, National Health Service; QALY, quality-adjusted life year

**Fig. 1:**
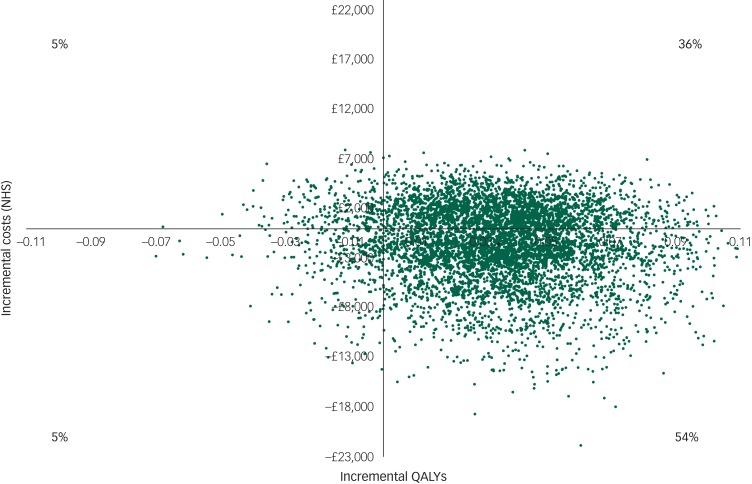
Cost-effectiveness plane (NHS perspective).

However, there was large uncertainty in the ICERs, led primarily by uncertainty in the costs. This is illustrated in the cost-effectiveness plane (Fig. 1), which displays the point estimate as a black bold dot and the uncertainty around the mean estimate as the surrounding ‘cloud’ of ICERs. This plane shows QALY gains at 90% certainty (i.e. 90% of the 10 000 bootstrapped ICERs were in the right half of the CE plane) and high uncertainty about the costs. The cost-effectiveness plane with costs from the societal perspective was very similar (Appendix 3).

The EVPI analysis showed that the population EVPI was approximately £87 million at a £0 ceiling ratio and decreased to approximately £33 million at an £85 000 ceiling ratio. This negative slope is explained by the negative ICERs resulted by the main analysis. The results of the EVPI analysis are presented in Appendix 4.

### Results of the sensitivity analyses

The results of the sensitivity analyses when using multiple imputation, propensity score matching or excluding medication costs were very similar to the results of the main analysis (Table 4). However, using regression analysis to adjust incremental costs and QALYs for their baseline values resulted in a 0.040 (95% CI 0.005 to 0.074) QALY gain, as well as £1319 (95% CI −412 to 4319) and £1579 (95% CI −410 to 5094) higher costs from the NHS and societal perspectives, respectively.

**Table 4:** Results of the sensitivity analyses

	CBT group	Standard care alone group	Incremental	ICER
	Mean (95% CI)	Mean (95% CI)	Mean (95% CI)	
*Imputed data-set*				
Costs: NHS perspective, £	3998 (1675 to 7431)	5529 (1424 to 13 717)	−1531 (−10 503 to 4712)	Dominant
Costs: societal perspective, £	4415 (1788 to 8284)	5650 (1523 to 13 843)	−1235 (−10 351 to 5332)	Dominant
QALYs	0.296 (0.255 to 0.336)	0.262 (0.233 to 0.292)	0.035 (−0.017 to 0.085)	
*Baseline adjustment*				
Costs: NHS perspective, £	2955 (1410 to 6173)	1636 (1037 to 2373)	1319 (−412 to 4319)	32 722
Costs: societal perspective, £	3324 (1529 to 6986)	1745 (1109 to 2536)	1579 (−410 to 5094)	39 187
QALYs	0.416 (0.380 to 0.453)	0.376 (0.336 to 0.413)	0.040 (0.005 to 0.074)	
*Propensity score matching*				
Costs: NHS perspective, £	3998 (1675 to 7431)	6674 (511 to 27728)	−2677 (−24255 to 5521)	Dominant
Costs: societal perspective, £	4415 (1788 to 8284)	6757 (619 to 27 793)	−2342 (−23 939 to 6166)	Dominant
QALYs	0.296 (0.255 to 0.336)	0.255 (0.208 to 0.300)	0.041 (−0.010 to 0.095)	
*Excluding medication costs*				
Costs: NHS perspective, £	£3318 (1288 to 6571)	5078 (993 to 13 253)	−1760 (−10 724 to 4349)	Dominant
Costs: societal perspective, £	3724 (1386 to 7368)	5199 (1092 to 13 396)	−1475 (−10 596 to 4930)	Dominant
QALYs	0.296 (0.255 to 0.336)	0.263 (0.236 to 0.291)	0.033 (−0.017 to 0.083)	

The results of each sensitivity analysis are based on 10 000 bootstrapped pairs of costs and QALYs; the sample size in all sensitivity analyses is the same as in the main analysis (i.e. 19 patients in the CBT group and 24 patients in the standard care alone group) except for the imputed data-set sensitivity analyses that included 24 patients in the CBT group and 26 patients in the standard care alone group.

CBT, cognitive behavioural therapy; NHS, National Health Service; QALY, quality-adjusted life year

**Fig. 2:**
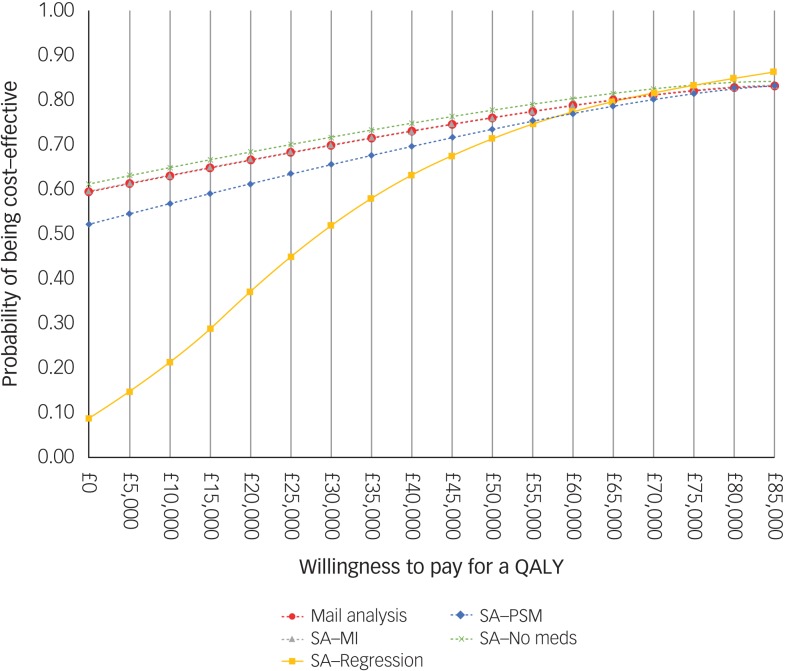
Cost-effectiveness acceptability curve and population EVPI. SA, sensitivity analysis; MI, multiple imputation.

For comparison purposes, the CEAC of the main analysis is presented alongside those of the sensitivity analyses in Fig. 2. According to the CEAC based on the main analysis, the probability of the CBT being cost-effective was 59% at a £0 ceiling ratio (i.e. the sum of 54 and 5% of 10 000 bootstrapped ICERs in the south-east and north-west quadrants of the cost-effectiveness plane, respectively) and increased to 83% at a £85 000 ceiling ratio. NICE suggests that the maximum acceptable ICER (i.e. ceiling ratio) is between £20 000 and £30 000 per QALY gained. Considering this range, the probability of the CBT treatment being cost-effective was 66% at a £20 000 ceiling ratio and increased to 70% at £30 000. The CEACs based on the sensitivity analyses were similar to those for the main analysis, except for the CEAC of the regression-based sensitivity analysis, which displayed less favourable results for the CBT at ceiling ratios lower than £50 000.

## Discussion

### Summary of main findings

This is the first economic evaluation of CBT for treating insomnia in people with schizophrenia. The results from the economic evaluation showed that the CBT delivered in the pilot BEST is likely to be more effective at lower costs in the short term compared with standard care alone. The results are subject to high uncertainty, driven primarily by large uncertainty in the costs. This might be explained by the small sample size and the large variation in costs among the patients included in the trial, as well as the study's short time horizon (i.e. 6 months post-randomisation).

Our results provide several indications that the CBT has the potential to be cost-effective (i.e. having an ICER between £20 000 and £30 000 or below). Although not statistically significant, the differences in costs between the two groups at baseline were considerable (i.e. £1462 NHS costs), favouring the standard care alone group. Even with this unequal staring point, patients in the CBT group had on average £1532 lower health and social care costs than patients in the standard care alone group during the follow-up period, indicating that CBT has the potential to be cost-effective. This potential is enhanced by the 90% certainty in the results for improvement in patient quality of life, which is expected to negatively affect costs in the longer term. In addition, we used very conservative assumptions when assessing the costs of the CBT intervention. For example, we used the upper range of additional contact time per treatment session (i.e. 20 min) and administration time per treatment session (i.e. 30 min). Relaxing these assumptions to be more realistic could also increase the likelihood of the CBT being cost-effective. The costs of productivity loss were not included in the analysis because there was only one person in employment in each group. However, the employment rate of people with schizophrenia in England is estimated to be between 5 and 15%.^14^ If the costs of productivity loss were included in the analysis and the sample was more representative in terms of employment, the likelihood of the CBT being cost-effective might potentially be improved, as positive changes in quality of life could yield higher participation in the labour market. Provision of sleep treatment also needs to be considered in the wider context of psychosis services. Patients want help for sleep problems, appreciate these concerns being taken seriously and value the provision of highly effective treatment.^15^ Positive experiences of the provision of sleep treatment are likely to enhance the take-up of effective treatments for other difficulties that the patients may experience.

The considerable uncertainty in the evidence available, however, warrants caution about the pilot results. Additional empirical information is needed to reduce further decision uncertainty (i.e. whether to provide CBT via the NHS). The results of the EVPI analysis suggest that the value of this additional information is high (approximately £87 million at a £0 ceiling ratio), given the large scale of the population affected by the decision (i.e. 100 000 patients with schizophrenia with sleeping disorders in England). A recent review concluded that economic evaluations with estimated EVPI values over £2 million were very unlikely to receive recommendations against further research.^13^ Hence, our results suggest that undertaking additional major commissioned research work, especially to collect more cost information, is necessary and worthwhile to further reduce the decision uncertainty.

### Challenges, strengths, and limitations

Similar to many pilot trials, the economic evaluation faced several challenges because of the small sample size of BEST. The five patients with missing observations comprised 21% of the patients in the CBT group, while the remaining 19 patients with complete observations in this treatment arm were too few to perform reliable imputation methods. Thus, a complete cases analysis was preferred in the main analysis, although this was valid and efficient only under the assumption that missing observations in the outcome variables (i.e. costs and EQ-5D-5L utilities in our study) are missing at random (i.e. the probability that data are missing does not depend on the values of the missing data). However, this might not be the case for the pilot BEST data, considering that non-completers were more severely affected patients than completers in the CBT group (see Appendix Table 2.4). Moreover, the small sample size may have contributed to the relatively large differences in the mean costs between the two trial arms at baseline. We performed PSM and regression analysis to reduce these differences, but their results should also be interpreted with caution owing to the limited statistical power. The economic evaluation had a short time horizon (6-month follow-up), which precluded assessment of the effects of CBT on costs and outcomes in the patient's lifetime. Poor-quality information on medication use was another limitation of the study.

The major strength of this study was the performance of several analyses that addressed the uncertainty in the results of the economic evaluation. Another strength was the value of information analysis, which showed substantial monetary benefits of further study of the cost-effectiveness of the CBT. To overcome the limitations of this study, future studies could recruit a larger patient population that is more representative, especially in terms of employment, and which is likely to have better-balanced costs at baseline. Future economic studies could also adopt a long(er) time horizon in the analysis and obtain good-quality medication data either by incorporating suitable questions in a survey or accessing administrative data. The influence of the CBT on productivity and state benefits could also be investigated in future studies.

### Implications for patients, clinicians and policy makers

Patients with schizophrenia have multiple complex health needs, as well as high rates of depression, suicidal ideation and poor physical health. The results of this study showed that treating pervasive sleep problems in this patient group with CBT is very likely to improve their quality of life in the short term. In the long term, such improvements could potentially reduce depression, psychotic experiences, irritability, exhaustion, drowsiness, reduced attention, poorer decision-making, cardiovascular disease, metabolic, abnormalities, weight gain, risk of type II diabetes and reduced immunity. Clinicians often use hypnotic medication to treat sleeping disorders. Clinical guidelines recommend CBT as the treatment of choice for chronic sleep difficulties. The results of this study indicate that CBT may be an effective and cost-effective intervention in this patient group. This alternative would also be aligned with patient preferences for psychological and behavioural-type therapies.^16^ If proven to be cost-effective, the CBT and associated training could be implemented in the NHS via the Improving Access to Psychological Therapies (IAPT) programme, which has been expanded for patients with severe mental illnesses such as schizophrenia. Furthermore, the need for greater access to psychological treatments for patients with schizophrenia is well recognised, and substantial investment has been suggested for this purpose.^17^ Efficiency is a crucial factor for the NHS in making wise investments while facing limited budgets, especially in mental healthcare. Our results indicate that CBT as provided in BEST is likely to be cost-effective, but further evaluation is necessary before stating whether it is good value for money or not.

### Conclusions

This study found that CBT for treating insomnia in people with schizophrenia is likely to be effective and cost-effective. A large trial designed to facilitate a thorough economic evaluation is needed to provide further evidence about its cost-effectiveness. This information is valuable and necessary to support decision makers in improving efficiency in mental healthcare in England.

## Appendix 2

**Table 2.1:** List of the unit costs and their sources

	Unit cost (£, 2014/15)	Source
GP contact	46	Unit costs of health and social care
GP home visit	91	Unit costs of health and social care
Night hospital admission	691	NHS reference costs 2014–15
Consultant	111	NHS reference costs 2014–15
Psychiatrist	124	NHS reference costs 2014–15
Social worker	40	Unit costs of health and social care
Counsellor	46	Unit costs of health and social care
Community psychiatric nurse	37	Unit costs of health and social care
Day care centre	35	Unit costs of health and social care
Day hospital	698	NHS reference costs 2014–15
Police contact	653	NAO analysis, based on CIPFA, Home Office, Ministry of Justice and Youth Justice Board Data.
Police cell	697	NAO analysis, based on CIPFA, Home Office, Ministry of Justice and Youth Justice Board Data.
Court	14 160	NAO analysis, based on CIPFA, Home Office, Ministry of Justice and Youth Justice Board Data.
National minimum wage per hour	6.50	GOV.UK (national minimum wage rates)

Medication	Median NHS indicative price per mg	Source
Amisulpride	0.004	BNF NICE (https://bnf.nice.org.uk/drug/)
Amitriptyline	0.004	BNF NICE (https://bnf.nice.org.uk/drug/)
Aripiprazole	0.229	BNF NICE (https://bnf.nice.org.uk/drug/)
Citalopram	0.007	BNF NICE (https://bnf.nice.org.uk/drug/)
Clonazepam	0.568	BNF NICE (https://bnf.nice.org.uk/drug/)
Clozapine	0.004	BNF NICE (https://bnf.nice.org.uk/drug/)
Depakote	0.001	BNF NICE (https://bnf.nice.org.uk/drug/)
Depixol	0.076	BNF NICE (https://bnf.nice.org.uk/drug/)
Diazepam	0.080	BNF NICE (https://bnf.nice.org.uk/drug/)
Fluoxetine	0.004	BNF NICE (https://bnf.nice.org.uk/drug/)
Haloperidol	0.054	BNF NICE (https://bnf.nice.org.uk/drug/)
Hydroxyzine	0.001	BNF NICE (https://bnf.nice.org.uk/drug/)
Lamotrigine	0.002	BNF NICE (https://bnf.nice.org.uk/drug/)
Lithium	0.000	BNF NICE (https://bnf.nice.org.uk/drug/)
Lorazepam	0.174	BNF NICE (https://bnf.nice.org.uk/drug/)
Mirtazapine	0.005	BNF NICE (https://bnf.nice.org.uk/drug/)
Olanzapine	0.016	BNF NICE (https://bnf.nice.org.uk/drug/)
Paliperidone	1.839	BNF NICE (https://bnf.nice.org.uk/drug/)
Paroxetine	0.004	BNF NICE (https://bnf.nice.org.uk/drug/)
Procyclidine	0.087	BNF NICE (https://bnf.nice.org.uk/drug/)
Promethazine hydrochloride	0.007	BNF NICE (https://bnf.nice.org.uk/drug/)
Quetiapine	0.005	BNF NICE (https://bnf.nice.org.uk/drug/)
Risperidone	0.106	BNF NICE (https://bnf.nice.org.uk/drug/)
Sertraline	0.009	BNF NICE (https://bnf.nice.org.uk/drug/)
Sodium valproate	0.001	BNF NICE (https://bnf.nice.org.uk/drug/)
Temazepam	0.010	BNF NICE (https://bnf.nice.org.uk/drug/)
Trazodone	0.006	BNF NICE (https://bnf.nice.org.uk/drug/)
Venlafaxine	0.005	BNF NICE (https://bnf.nice.org.uk/drug/)
Zopiclone	0.010	BNF NICE (https://bnf.nice.org.uk/drug/)
Zuclopenthixol decanoate	0.015	BNF NICE (https://bnf.nice.org.uk/drug/)

NHS, National Health Service; NAO, National Audit Office; CIPFA, Chartered Institute of Public Finance and Accountancy; BNF, British National Formulary; NICE, National Institute for Health and Care Excellence.

**Table 2.2:** Pattern of missing observations

Patient	Treatment arm	Baseline		3-month follow-up		6-month follow-up	
		Costs	EQ-5D-5L utility	Costs	EQ-5D-5L utility	Costs	EQ-5D-5L utility
1	CBT			X	X		X
2	CBT		X				
3	CBT			X	X	X	X
4	CBT					X	X
5	CBT					X	X
6	Control			X	X	X	X
7	Control			X	X	X	X

X indicates a missing observation.

CBT, cognitive–behavioural therapy

**Table 2.3:** Intervention costs

	Mean (s.d.)
Total treatment sessions per patient	6.71 (1.86)
Treatment sessions at home per patient	3.79 (3.19)
Session duration (minutes)	60
Additional contact time per treatment session (minutes)	20
Administration time per treatment session (minutes)	30
Travel time per home visit (minutes)	94.29 (45.02)
Total labour time (minutes)	1393.57 (549.27)
Hourly rate of psychologist	£20.27
Labour costs per treated patient	£470.79
Mean mileage per visit	42.29 (27.48)
Cost per mile	£0.45
Travelling costs per treated patient	£19.03
Total costs per treated patient	£490.00

**Table 2.4:** Differences at baseline between completers and non-completers

Measurements at baseline	CBT		Control	
	Completers *n* = 19	Non-completers (*n* = 5)	Completers (*n* = 24)	Non-completers (*n* = 2)
	mean (s.d.) median [min–max]	mean (s.d.) median [min–max]	mean (s.d.) median [min–max]	mean (s.d.) median [min–max]
Age	40.4 (12.7) 42.0 [18–65]	36.6 (6.6) 39.0 [26–43]	42.0 (13.4) 46.5 [18–64]	32.5 (14.8) 32.5 [22–43]
EQ-5D-5L utility	0.58 (0.21) 0.61 [0.11–0.88]	0.52 (0.19) 0.51 [0.34–0.73]^a^	0.60 (0.22) 0.64 [0.08–1]	0.61 (0.29) 0.61 [0.41–0.82]
Total NHS costs	7972 (15 731) 1745 [295–60 049]	24 568 (18 241) 20 625 [1734–52 195]	6510 (13 272) 1912 [285–48 985]	680 (346) 680 [436–925]
Total societal costs	8126 (15 740) 1745 [314–60 049]	25 109 (18 293) 21 297 [2387–52 887]	7071 (13 311) 2136 [426–49 076]	680 (346) 680 [436–925]

The *t*-tests and Mann–Whitney tests showed no statistically significant differences between completers and non-completers in either of the treatment arms.

CBT, cognitive–behavioural therapy; NHS, National Health Service

a. *n* = 4 because a non-completer in the CBT group had no EQ-5D-5L measurement at baseline.
